# Increased COVID-19 Lockdown Burden in Italian Adults with Gastrointestinal Diseases

**DOI:** 10.3390/nu13061820

**Published:** 2021-05-27

**Authors:** Monica Ruotolo, Mario Gagliardi, Carolina Ciacci, Fabiana Zingone, Corina de Santis Ciacci, Antonella Santonicola, Giovanna D’Arcangelo, Monica Siniscalchi

**Affiliations:** 1 Department of Medicine, Surgery, Dentistry, Scuola Medica Salernitana, University of Salerno, 84084 Fisciano, Italy; monicaruotolo@inwind.it (M.R.); mariogagliardi@outlook.com (M.G.); antonellasantonicola83@gmail.com (A.S.); msiniscalchi@unisa.it (M.S.); 2 Gastroenterology Unit, Department of Surgery, Oncology and Gastroenterology, University of Padua, 35122 Padua, Italy; fabiana.zingone@outlook.com; 3 School of Medicine, University of Naples Federico II, 80138 Napoli, Italy; carolina.ciacci@sangiovannieruggi.it; 4 Department of Systems Medicine, University of Tor Vergata, 00133 Rome, Italy; giovanna.darcangelo@uniroma2.it

**Keywords:** COVID-19, lockdown, lifestyle, emotional balance, Italy, dietary habit

## Abstract

Background: Coronavirus disease 2019 (COVID-19) causes not only severe illness but also detrimental effects associated with the lockdown measures. The present study aimed to evaluate reported lifestyle changes in a cohort of adults in Italy, including physical exercise, food choices, and psychological wellbeing, after two months of lockdown. Methods: A web survey on social media (Facebook and LinkedIn) of 32 multiple-choice questions aiming to evaluate the impact of the national COVID-19 lockdown in a sample of Italian adults. Results: We received 1378 complete responses (women 68.3%, mean age 39.5 ± 12.5 years). The percentage of participants reporting regular exercise decreased during lockdown (52 vs. 56.5%). The vast majority of people continued to consume the three traditional meals per day, but the consumption of meat, fish, and eggs significantly decreased. Women reported more frequent anxiety, sadness, fear, and feelings of insecurity than men. The factors predicting the worst outcome during the lockdown were being a woman, low education and income, gastrointestinal diseases. Conclusion: The lockdown has had a limited impact on food choices and physical exercise in Italian adults of our series, since most of them made an effort to improve their lifestyle. However, women with gastrointestinal diseases reported more frequent negative feelings and poor adaptation to the lockdown.

## 1. Introduction

Coronavirus disease 2019 (COVID-19) is an emerging disease caused by a virus phylogenetically in the SARS-CoV-2 [1]. Italy was the first country in Europe to experience the outbreak, with a rapid spread of infection. On 21 February 2020, the Italian Government locked Lombardy and Veneto (Northern Italy), followed a few days later by a strict nation-wide lockdown. Schools, offices, and all non-essential shops closed down. Only one person per household per day was allowed to leave the house wearing a protective mask, and only for necessary journeys unless working in essential services. A recent report suggests that the SARS-CoV-2 pandemic may have affected psychological behavior and mental health worldwide due to the imposed social distancing [2]. Notorious images of empty shelves in food stores and long lines of customers reflected the fear of being left with no food in the household [3]. The COVID-19 pandemic changed lifestyle habits, especially physical exercise, nutrient intake, and food choices. A recent systematic review reported high frequency of food intake, a large number of main meals, and consumption of snacks and unhealthy foods during the COVID-19 pandemic [4]. Lifestyle change has been a matter of concern for many people, as Italians are conscious of a healthy lifestyle through consulting primary healthcare and specialists [5].

An essential aspect of the recent experience is the psychological impact of the lockdown, which imposed physical limitations on the Italian population [6]. The lockdown effect might also have been notable in changing health-related behaviors, such as eating habits [7]. The relationship between emotions and food is a bilateral relationship: what we eat affects our mood, and at the same time, the feelings we experience change our way of eating [8].

The present survey aimed to investigate how people changed food habits during the lockdown and how the COVID-19 pandemic has affected their emotions and food consumption.

## 2. Materials and Methods

We used a web survey that included 32 multiple-choice questions aiming to evaluate the impact of the COVID-19 pandemic on the food choices and emotions of a sample of Italian adults (see Supplementary File). The homepage of the survey provided information on the scope and purpose of the study. The participants read and agreed with the use of the data information provided. The survey was disseminated through institutional and private social networks (Twitter, Facebook, and Instagram), and institutional mailing lists between 24 April and 8 May 2020, which corresponds to 73 (Veneto and Lombardy) and 63 (rest of Italy) days of strict measures of the lockdown in Italy. Lombardy, Liguria, Emilia Romagna, and Veneto, at the time of the questionnaire, were considered ‘red zones’ with a high frequency of infection and high mortality rate from COVID-19. Campania, Tuscany, Lazio, Marche, although in preventative lockdown, were considered ‘orange zones’, with a lower infection prevalence and lower mortality rate. Finally, Basilicata, Puglia, Calabria, Sicily, and Sardinia were the regions with the lowest contagion; we define them here as ‘yellow zones’. We also collected data on weight gain, working status, physical exercise pre- and post-lockdown and the presence of any comorbidities [9]. The present study reports a selection of the data collected.

### Statistical Analysis

Categorical variables were expressed as frequency and continuous variables as mean ± standard deviations (SD). Comparisons among categorical and continuous variables were performed using the chi-square test and *t*-test, respectively. All analyses were two-tailed with a significance level set at *p* < 0.05. We analyzed the data using STATA 11 software (Stata Corp., College Station, TX, USA).

## 3. Results

### 3.1. Demographics

We received 1378 complete responses out of 1408 responders. The vast majority of respondents reported staying at home for the lockdown measures from 24 February to 8 May (82%). Table 1 shows the demographic characteristics of all participants. The majority of respondents were women (937, 68.3%); three people did not identify with a specific gender (0.3%) and were excluded from the analysis. At the time of the survey, the mean age was 39.5 ± 12.5 years, with most subjects aged between 30 and 49 years (48.7%). The majority of respondents lived in the orange regions (56.1%), but we found no differences in sex, age, type of work, and anthropometry between red, orange, and yellow zone participants. Among participants, 118 (8.6%) respondents defined themselves as not working at all (likely homemakers or unemployed); more women than men were unemployed (7.3% vs. 1.3% *p* = 0.001). However, more women than men reported being professionals or having a white-collar job (13.1% vs. 5.7% *p* = 0.01). More people living in the red zone reported having a job than in the orange zones (*p* = 0.05). Only about 21% of respondents reported working as usual (those employed in essential services, mostly healthcare, food supply, and retail). However, 536 people (38%) declared that they did not work during the lockdown.

Respondents reported suffering from a disease in 36.6% of cases, and from gastrointestinal illness in 13.2%. Table 2 shows the reported health problems and illnesses according to gender: cardiovascular diseases, liver diseases, and diabetes are more frequent in men, while reported functional gastrointestinal and mood disorders were more frequent in women.

The distribution of the reported diseases was not different among zones of residence (data not shown).

### 3.2. Physical Exercise during the Lockdown

Seven-hundred-and-seventy-eight participants (56.5%) reported exercising regularly before the lockdown. The number slightly decreased during lockdown (52%); interestingly, pre-lockdown, there was not a statistically significant difference between sex and age in exercise, while during the lockdown, fewer young people continued to exercise at home (*p* < 0.001). Before lockdown, persons living in red regions reported habitual physical exercise less frequently than persons in non-red regions (8.3% vs. 48.1%, *p* = 0.003). Post lockdown, persons from both red regions and non-red regions reduced their habitual physical activity (7.3% vs. 44.7%, *p* = 0.013), suggesting that the lockdown and stay-at-home policy was associated with changes in reported physical activity. In univariate logistic regression, variables associating positively with leisure physical activity during lockdown were lower age (*p* < 0.001), habitual leisure physical activity pre-lockdown (*p* < 0.001), blue-collar/farmer occupation (*p* = 0.033), and a higher score of positive emotions (*p* = 0.012). In multivariate analysis, independent negative associations were found for the male sex, older age, lack of leisure physical activity pre lockdown, greater occupation, and a score of negative emotional status (*p* < 0.032; coefficients not shown).

### 3.3. Weight Gain and Food Choices

The Body Mass Index (BMI) of respondents at the time of the survey was 24.95 ± 4.6 kg/m^2^ (women: 24.3 ± 4.1, men: 26.4 ± 5.2, *p* < 0.001). The vast majority of people reported that they were still having three meals per day even if they stayed at home.

Table 3 summarizes data relative to the reported weight gain. Seven-hundred-and-seventeen adults (52.03%) reported weight gain after about 60 days of lockdown. The majority reported a weight gain between 1 and 2 kilograms (kg). No differences in weight gain were noted among women and men and those living in the three COVID-19 risk zones. A statistically significant difference in weight gain exists, instead, among age groups. The majority of patients of age groups 30–49 and 50–69 reported a weight gain of 1–2 kg (Table 3).

More than 45% of respondents reported drinking more than five glasses of water per day, while only 3.2% reported drinking two or fewer; no difference was noted among sexes (*p* = 0.626). Eighty-six percent of participants never bought food on the internet, about 5% occasionally, and 9% regularly. However, women bought food on the internet more frequently than men, although the finding did not reach a statistical significance (regularly shopping for food on the internet 5.4% vs. 3.7%, *p* = 0.071). Women reported more frequent regular home cooking than men (64.7% vs. 28.7%, *p* = 0.029).

There was no difference among the three COVID-19 risk zones regarding the type of food and beverages chosen (data not shown). Therefore, data are presented for the whole population. Table 4 shows the reported percentage frequency of specific food picks per week. Table 5 shows the comparison of the reported food and beverage intake of the present survey with the 2019 data from the National Institute of Statistics for age-matched respondents. Data show that Italians in our sample significantly reduced their consumption of red meat, pork and preserved meat, poultry, fish (fresh fish was hard to find), eggs. Italians also reported a borderline significant reduction of sweets (*p* = 0.08) [10]. Our survey respondents reported a statistically significant lower habitual intake of soft drinks during the lockdown.

In univariate logistic regression, variables associating positively with weight gain during lockdown were leisure physical activity before and during lockdown (*p* = 0.020 and <0.001, respectively), intestinal disease (*p* = 0.024), and score of negative emotional status (*p* = 0.011). In multivariate analysis, independent associations were found only for leisure physical activity during the lockdown (*p* = 0.002) and intestinal disease (*p* < 0.001, coefficient not shown).

### 3.4. Emotional Impact

**Figure 1:**
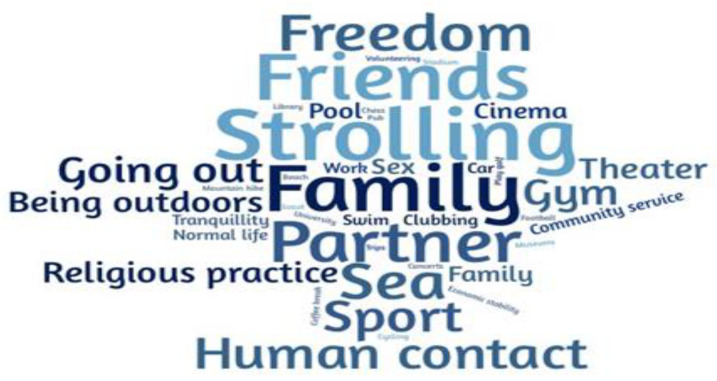
Word cloud representation of the main answers to the question: In a word, what do you miss more?

**Figure 2:**
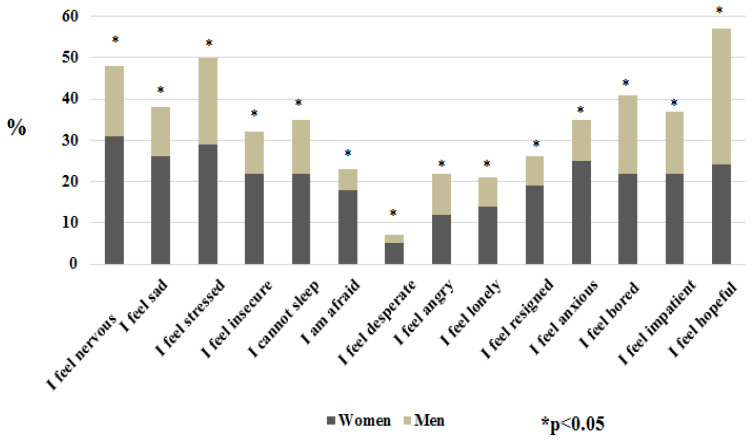
Percentages of patients reporting “very much” for specific feelings by gender. The reported feelings were significantly more frequent in women than men (* *p* < 0.05).

Figure 1 shows a word cloud for the words that the respondents wrote in the free text box regarding the question: “In a word, what do you miss more?” Respondents most frequently wrote strolling (in Italian *passeggiare*, i.e., to take a walk with no definite purpose), followed by the sea, freedom, partner, and human contact. Figure 2 shows that anxiety, sadness, fear, and feelings of insecurity were significantly more frequent in women than men. The majority of respondents reported being nervous, scared, anxious, irritated, resigned, and lonely. No difference was noted between women and men for the feeling of being impatient, apathetic, staying well, satisfied, grateful, happy, and joyful. Almost 55% of respondents stated that none of the reported feelings influenced their eating behaviour, 35.34% were somewhat influenced, and 10% were influenced. However, the gender comparison revealed that 49% of women and 67% of men reported no influence of emotional states on their food choices (*p* < 0.001).

In univariate linear regression, variables associated with a higher score of negative emotional status were female sex (*p* < 0.001), older age (*p* < 0.001), lower working class (*p* < 0.001), report of any disease (*p* < 0.001), report of intestinal disease (*p* < 0.001), and lower score of positive emotions (*p* < 0.001). In multivariate analysis, independent associations were found for sex, age, leisure physical activity during lockdown, working class, report of any disease, report of intestinal disease, and score of positive emotions. In univariate linear regression, variables associated with higher score of positive emotions were lower age (*p* < 0.001), home with garden/terrace (*p* = 0.013), leisure physical activity during lockdown (*p* = 0.012), and lower score of negative emotional status (*p* < 0.001). In multivariate analysis, independent associations were found for age (*p* = 0.001), home with garden/terrace (*p* = 0.011), and score of negative emotional status (*p* < 0.001; coefficient not shown).

## 4. Discussion

Our study shows that the unique circumstance of a global pandemic determined a massive change in Italian lifestyle. Only about 20% respondents of the survey were allowed to go out for regular work, the rest having sensibly reduced their working activity or stopped working. However, women reported overall greater burden due to the lifestyle change than men. Furthermore, women with gastrointestinal diseases reported more frequent psychological imbalance.

However, the unexpected finding of our survey is that the majority of respondents reported taking better care of themselves with wiser food and drink choices and maintaining some physical exercise.

Respondents reported that during the lockdown, they tended towards a healthy lifestyle even if they did not suffer any of the diseases considered at high risk of mortality from COVID-19 [11]. We compared the consumption of some foods of our population with the ISTAT-reported general population consumption for the year 2019. The analysis showed that during the lockdown period, there was a significant reduction in the consumption of meat, eggs, fish, dairy products, and soft drinks compared to the average ISTAT values. Instead, the consumption of foods such as vegetables, fruit, pasta, and bread did not significantly vary. One possibility is that during the lockdown people have been more careful in choosing food, favoring those with the best nutritional value. The only exception was for alcoholic beverages, for which the consumption remained unchanged compared to ISTAT data. In our sample, however, men increased their alcohol intake more than women. It is also possible that for a proportion of respondents that were used to eating out, home cooking was just basic and included mostly pasta and vegetables. The last possibility is that the consumption of meat, fish, and dairy products decreased because these items are more expensive than pasta and vegetables. The use of eggs was also reported to be lower than in the ISTAT 2019 age-matched sample. Given that our respondents reported a healthy diet, we can conclude that the modest weight gain reported is likely due to the reduction in physical exercise. Moreover, a recent review evaluating studies revealed that stress and anxiety might be associated with higher or lower weight status [12]. Women compared to men reported a higher frequency of emotional imbalance due to reported nervousness, anxiety, and fear. Our findings are in line with a recent study reporting a more significant psychological impact of the outbreak and higher levels of stress, anxiety, and depression in women than in men [13], despite male respondents being overall less fit than women in our sample, and therefore at higher risk of a more severe COVID-19 infection [14].

In consideration of the demographics, nutritional and emotional data, our survey data indicate that mainly women with a medium–high education oriented their choices towards healthy food, mostly home-cooking their meals (or for the whole family) during the period of quarantine. Women reduced the frequency of consumption of food with a high calorie content, and did not increase alcohol consumption despite the increased emotional burden of the pandemic.

Our findings are only partially in keeping with a recent Italian study that described that the majority of individuals from the general population reported no likelihood of psychological distress [2].

The findings of the present study allow a few hypotheses. First, respondents led a healthy lifestyle for fear of getting sick or because of the sense of uncertainty and insecurity. Secondarily, the possibility of smart-working but also the increased burden of household chores (cooking, cleaning, looking after children, assisting elderly parents) prompted people to consume simple, healthy meals. Another possibility is that the impossibility of eating outside in restaurants and pubs limited the number of unhealthy food choices. The last possibility is the economic one. Many people feared for the future, some lost their jobs or were laid off. The cost of some food, such as meats, fresh fish, imported cheeses, increased. At the same time, in most supermarkets, the prices of basic Italian meals (pasta, bread, vegetables, fruits) decreased because of the policy of supporting people in need during the lockdown.

Our study has some strengths. The COVID-19 experience has confirmed that online surveys are a useful tool in tracking the public perception, mainly by using social media platforms, during disease outbreaks [15]. In our experience, surveys also allowed us to gather helpful information to improve the healthcare of patients [16,17].

The present survey is the first to report on Italian lifestyle habits during the lockdown for COVID-19, reaching the respondents after two months of lockdown when they were still at home and there was no certainty of an end in sight. The number of respondents and their gender and occupation distribution is adequate to obtain accurate information about perceptions and lifestyle habits.

Our study also has some limitations. We selected respondents by posting the survey on social platforms. Thus, we might have a selection bias. However, the two platforms used allowed us to reach in a quite limited period different types of people: students, homemakers, low-income workers, retired people but also professionals. We cannot exclude, although it is unlikely, that those who did respond were those most careful about their lifestyle even in the current situation [18]. However, the composition of our population sample encompasses women and men, different ages, different types of work and economic levels. Lastly, we did not utilize a validated questionnaire, but questions formulated ad hoc for the COVID-19 lockdown circumstance. The survey included open questions and multiple-choice answers in order not to influence the responses of respondents.

A recent systematic review of data from seven studies reported an overall increase in food consumption, weight, body mass index (BMI), and a change in eating style. Overall, the studies suggest that healthy nutrition may be affected by preventive measures to restrict physical contact as a result of the COVID-19 syndemic [5]. Furthermore, in a large online survey in the UK, a large number of participants reported negative changes in eating and physical activity behavior and experiencing barriers to weight management compared to before lockdown [19]. Another recent survey conducted in the United States reported that about 73% of participants experienced moderate to high levels of perceived stress that significantly correlated with emotional eating [20].

However, our findings were in line with other recent studies conducted in Italy and in other countries [21,22]. In a French survey [21], although the nutritional quality of diet was lower during the lockdown compared to before, there was an increase in the importance of weight control that was associated with increased nutritional quality. Di Renzo et al. [22] reported the results of a large Italian survey and described a modest weight gain and an increase of homemade recipes (e.g., sweets, pizza, and bread), cereals, legumes, white meat, and hot beverage consumption, and a decrease of fresh fish, packaged sweets, and baked products, delivery food, and alcohol intake. Different from our study, no comparison was made with known intakes of food from the ISTAT data.

In conclusion, the results of our survey demonstrate that the lockdown had a limited impact on the food choices and physical exercise of Italian adults. Moreover, despite the high frequency of apprehension, the majority of respondents, and mostly women, made an effort to improve their lifestyle, eat well, and exercise at home.

## Figures and Tables

**Table 1:** Demographics and selected characteristics of the respondents.

Variables	Participants *N* = 1375 (100%)
**Gender**	
Women	937 (68.2%)
Men	438 (31.8%)
**Age Groups, years (%)**	
18–29	169 (12.3%)
30–49	669 (48.7%)
50–69	490 (35.6%)
>70	47 (3.4%)
**During the lockdown living with (%)**	
Family	1215 (88.4%)
alone	17 (1.2%)
other	143 (10.4%)
**Region of residence (%)**	
Red zone	171 (12.4%)
Orange zone	773 (56.2%)
Yellow zone	431 (31.4%)
**Type of work**	
Unemployed	118 (8.6%)
Students	107 (7.8%)
Retired	26 (1.9%)
Workers	1124 (81.7%)
**Are you regularly going to work?**	
I regularly go to work	237 (21.1%)
I go to work fewer days than before	138 (12.3%)
I am in smart working at home	433 (38.5%)
I am not going to work, nor do I work at home.	316 (28.1%)
**The house in which you are living has a:**	
Garden	435 (31.6%)
Balcony	547 (39.8%)
Window	115 (8.3%)
Common open space	278 (20.3%)

In univariate logistic regression, variables associated positively with working during lockdown were male sex (*p* = 0.002), older age (*p* < 0.001), and a lower score of negative emotional status (*p* < 0.001). These associations were independent and significant in multivariable analysis.

**Table 2:** Percentage of reported morbidities of the respondents, also according to gender.

	Total *N*= 1375 (100%)	Women *N* = 937	Men *N* = 438	*p* *
Dyslipidaemia	15.7	14.3	18.5	0.05
Cardiovascular	13.8	10	22.1	**<0.001**
Liver	0.5	0.2	1.1	**0.02**
Functional GI disorders	11.3	13.1	7.5	**0.002**
Inflammatory bowel disease	1.9	1.5	2.7	**0.1**
Diabetes	2.8	1.7	5.2	**<0.001**
Food intolerance/allergies	1.4	1.8	0.7	**0.1**
Mood disorders	9.2	10.9	5.5	**0.001**
Other	12.6	15.1	7.3	**<0.001**

* The *p*-values that are statistically significant are in bold.

**Table 3:** The reported weight gain during the 60-day lockdown.

	Female	Male	Participants: 1375 (100%)	*p* *
**Reporting weight gain**	492 (52.51%)	222 (50.68%)	714 (51.93%)	0.52
**Age groups reporting weight gain**				0.73
18–29	54 (44.63%)	28 (58.33%)	82 (48.52%)	
30–49	255 (52.04%)	91 (50.84%)	346 (51.72%)	
50–69	172 (56.21%)	90 (48.91%)	262 (53.47%)	
>70	11 (55%)	13 (48.15%)	24 (51.06%)	
**Reporting weight gain**				0.12
Red zone	55 (46.22%)	22 (42.31%)	77 (45.35%)	
Orange zone	293 (55.39%)	123 (50.41%)	416 (53.82%)	
Yellow zone	144 (49.83%)	77 (54.23%)	221 (51.50%)	
**Average weight gain in kg**				**0.045**
1–2	351 (63.93%)	156 (61.9%)	507 (63.3%)	
3–4	150 (27.32%)	78 (30.95%)	228 (28.46%)	
>4	48 (8.74%)	18 (7.14%)	66 (8.24%)	

* The *p*-values that are statistically significant are in bold.

**Table 4:** The reported weekly intake of specific foods. (F: Female; M: Male).

Foods	Never % F/M	Once a Week % F/M	2–3 Times a Week % F/M	Every Day % F/M
Red meats	11.9/6.1	51/52	37/41	0.1/0.9
Poultry	7/7	38/41	53/51	2/1
Pork/preserved meat	19/12	51/47	28/38	2/3
Eggs	5/4.8	63/66	30/27	1/2
Fish	9/9	49/52	38/37	4/2
Dairy products	28/32	20/21	29/26	23/21
Cereals (wheat/other grains)	3/3	9/8	32/30	56/59
Vegetables	1/1	3/7	25/46	71/46
Beans	10/6	47/44	41/48	2/2
Fresh fruit	6/5	6/7	23/26	65/62
Dried fruits and nuts	33/33	23/30	25/23	19/14
Salty snacks	7/8	58/48	30/38	5/6
Sweets (candy bars, cakes, croissant, etc.)	6/6	27/26	37/40	30/28
Fruit juices	83/72	7/15	7/8	3/5
Sodas	67/59	20/23	9/13	4/5

**Table 5:** The percentage of reported consumption of selected foods and beverages of the population according to the Italian Institute of Statistics (ISTAT) data 2019 versus the present survey data 2020 for the quantity indicated by responses of “a few times a week”.

Variable	ISTAT 2019	Survey 2020	*p* *
Red meat	53	38	**0.03**
Poultry	79	53	**<0.001**
Pork/preserved meat	60	31	**<0.001**
Eggs	66	30	**<0.001**
Fish	62	37	**<0.001**
Dairy products	21	8	**0.01**
Beans	54	44	0.15
Salty snacks	28	32	0.53
Sweets	50	38	0.08
Soft drinks	61	36	**0.000**
Wine	56	53	0.886
Beer	54	47	0.322
Spirits	34	25	0.162

* The *p*-values that are statistically significant are in bold.

## Supplementary Materials

The following are available online at https://www.mdpi.com/article/10.3390/nu13061820/s1. Web-based survey model.
