# Supplementary material for: Increased COVID-19 Lockdown Burden in Italian Adults with Gastrointestinal Diseases
Source: Nutrients. 2021 May 27;13(6):1820. doi: 10.3390/nu13061820 (PMC8230132; doi:10.3390/nu13061820)
Supplement: Supplementary file 1 [file nutrients-13-01820-s001.zip › nutrients-1187253-supplementary.pdf]

APPENDIX 1

Dear Participant,

The Gastroenterology Unit of the University of Salerno is conducting a study on lifestyle changes and emotional balance during the COVID 19 pandemic. The questionnaire is anonymous and will take only a few minutes. The information in this study will be used only for research purposes and in ways that will not reveal who you are, according to Italian law (art. 13 UE Regulation n.679/2016 del 27.04.2016 and D.Lgs. 30 June 2003 n. 196)

I declare that I have read the information and authorize the processing of data*

- Yes
- No

- **Sex**
- Male
- Female

- **Where are you living in this period?**
  - At home, with my family
  - At home, with my friends
  - Alone
  - Residential care facility

- **In which Italian Region are you living now?**

- Valle d'Aosta
- Piemonte
- Liguria
- Lombardia
- Veneto
- Trentino Alto Adige
- Friuli Venezia Giulia
- Emilia Romagna
- Toscana
- Umbria
- Marche
- Lazio
- Abruzzo
- Molise
- Campania
- Puglia
- Basilicata
- Calabria
- Sicilia
- Sardegna

- **What is your age range?**
  - 18 – 29years
  - 30 – 49years
  - 50 – 69years
  - 70 – 79years
  - 80 years or more

- **Weight Kg ___________**

- **Height cm____________**

Did you gain weight during the lockdown?

- Yes
- No

If yes, how many Kilograms?

- 1-2 kg
- 3-4 kg
- More than 4kg

What is your job?

- Unemployed
- Student
- Retired
- Worker

Have you beengoing to workregularly during this period?

- Yes, I regularly go to work
- Yes, butI go to work fewer days than before
- I am in smart working at home
- I am not going to work, nor do I work at home.

When did you start staying at home because of the COVID 19 pandemic?

- Before 24thFebruary
- From 24th February to 1st March
- From 2ndMarch to 8th March
- From 9th March to 15th March
- From 16th March to 22th March
- From 30th March to 5th April
- I rarely went out even before the pandemic.
- I have been going out regularly in this period

Did you perform physical exercise before the lockdown?

- Yes
- No

Are you doing physical exercise at home in this period?

- Yes
- No

If yes, what type of physical exercise?

- Mild
- Moderate
- Intense

How often?

- Everyday
- 3 times a week
- Rarely

What do you miss more?

- Freedom
- Friends
- Family
- Partner
- Sea
- Sport, gym
- Human Contact
- Going out
- Religious Practice

Do you have one of these health problems?

- Dyslipidaemia
- Cardiovascular disease
- Liver diseases
- Functional Gastrointestinal disorders
- Inflammatory bowel disease
- Diabetes
- Food intolerance /allergies
- Mood disorders
- Other
- None

During the lockdown, your health problem…

- Improved
- Got worse
- Remained the same

Do you usually have breakfast?

- Yes
- No
- Sometimes

Do you usually have lunch and dinner?

- Yes
- No
- Sometimes

How many meals do you have per day?

- 1
- 2
- 3
- 4
- 5 or more

How do you manage your meals?

- I cook at home the food I bought in the supermarket
- I eat what is delivered to me at home
- I order take away food

Did you buy food fromthe internet?

- Yes
- No
- Sometimes

Did you take any supplements during this period?

- Yes
- No
- Sometimes

How many glasses of water do you think you drank per day in this period?

- 1
- 2
- 3
- 4
- 5 or more

How often did you eat these foods?

Foods	Never	Once a week	2-3 times a week	Every day
Redmeats				
Poultry				
Pork /preservedmeat				
Eggs				

Fish				
Dairyproducts				
Cereals (wheat/ othergrains)				
Vegetables				
Beans				
Freshfruit				
Driedfruits and nuts				
Saltysnacks				
Sweets (candy bars, cakes, croissant etc.)				
Fruitjuices				
Sodas				

What were your feelings during the lockdown?

	**Notatall**	**Much**	**Verymuch**
I feel nervous			
I feel sad			
I feel stressed			
I feel insecure			
I feel irritated			
I cannot sleep			
I feel apathetic			
I feel impatient			
I am afraid			
I feeld esperate			
I fee langry			
I feel lonely			
I feel resigned			
I feel anxious			
I feel bored			
I feel well			
I feel satisfied			
I feel hopeful			
I feel grateful			
I feel happy			
I feel joyful			
I feel excited			

How do these feelings impactyour diet?

- o Not at all
- o Much
- o Very much

Did you have sleep disorders during this period?

- No or rarely
- Sometimes
- Often

The house in which you are living has a:

- Garden
- Balcony
- Window
- Common open space
